# FTO deficiency facilitates epithelia dysfunction in oral lichen planus

**DOI:** 10.1016/j.omtn.2025.102463

**Published:** 2025-01-25

**Authors:** Yufeng Fan, Yukai Hao, Yan Ding, Xiangyu Wang, Xuejun Ge

**Affiliations:** 1 Shanxi Province Key Laboratory of Oral Diseases Prevention and New Materials, Shanxi Medical University School and Hospital of Stomatology, Taiyuan, Shanxi, China; 2 Department of Endodontics, Shanxi Medical University School and Hospital of Stomatology, Taiyuan, Shanxi, China; 3 Department of Dermatology, Hainan Provincial Hospital of Skin Disease, Haikou, Hainan, China; 4 Department of Dermatology, Skin Disease Hospital of Hainan Medical University, Haikou, Hainan, China

**Keywords:** MT: RNA and epigenetic editing Special Issue, fat mass and obesity-associated protein, oral lichen planus, vitamin D receptor, RNA m^6^A modification, oral epithelial dysfunction

## Abstract

The fat mass and obesity-associated protein (FTO) is identified as regulating mammalian development and diseases by removing methyl groups from RNAs. However, the roles of FTO in the context of oral lichen planus (OLP) remain unknown. Here, we demonstrated that the protein levels of FTO in the keratinocytes from OLP patients were down-regulated compared to those from healthy participants. At the molecular level, we explained that GSK-3β-induced phosphorylation promoted FTO protein degradation in diseased oral keratinocytes. Using a cell co-culture model, we further confirmed that FTO deficiency facilitated NF-κB activation and apoptosis in oral keratinocytes under inflammatory conditions. Vitamin D receptor (VDR), which plays a protective role in OLP, was mediated by FTO in an RNA *N*^6^-methyladenosine (m^6^A) methylation-dependent way. FTO overexpression failed to suppress NF-κB and caspase-3 activities upon VDR ablation in oral keratinocytes, suggesting that FTO insufficiency damages oral epithelial by targeting VDR. Collectively, these data reveal that FTO deficiency facilitates epithelial dysfunction in OLP by decreasing VDR expression.

## Graphical abstract

**Figure:**
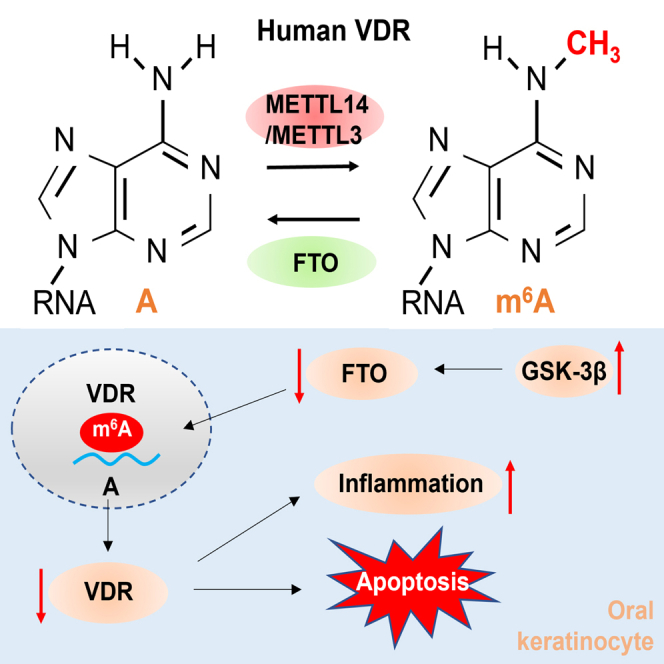

## Teaser

RNA m^6^A demethylase FTO plays critical regulatory roles in oral lichen planus development. Targeting FTO might be a promising therapeutic approach to prevent or manage oral lichen planus.

## Introduction

Oral lichen planus (OLP) is known as a mucocutaneous inflammatory disorder with approximately 1% prevalence across the globe.^1^ The severity of OLP ranges from mild to moderate to severe conditions, which undergoes quiescence and exacerbation periods.^2^^,^^3^ The clinical symptoms of OLP often coincide with stress, trauma, anxiety, or exposure to chronic irritants like dental fillings, tobacco, or dental plaque.^2^^,^^3^ Although the etiology of OLP remains elusive, oral mucosal immune system dysregulation such as massive T lymphocyte infiltration mainly contributes to the onset of this disease.^4^ The critical histopathological characteristics of OLP are a dense inflammatory T cell band in the subepithelial area as well as intra-epithelial lymphocytic cell invasion and degeneration in the basal epithelial layer.^5^ In addition, some histological features of OLP such as surface hyperparakeratosis and a fibrinous precipitate were observed.^6^ To date, the overall consensus of OLP pathogenesis is that the recognition of non-self-antigens triggers a complicated interaction between immune and epithelial cells, cytokines and adhesion-related molecules, ending up with an uncontrolled immune response that results in basement membrane destruction and keratinocyte apoptosis.^4^

*N*6-Methyladenosine (m^6^A) is identified as being the most prevalent chemical modification in mammalian RNAs that affects stability and translation efficiency of mRNAs.^7^^,^^8^^,^^9^ The methyl groups on RNAs are transferred by methyltransferases, recognized by reader proteins and removed by demethylases.^7^^,^^8^^,^^9^^,^^10^ The fat mass and obesity-associated protein (FTO) is the first demethylase related to mRNA m^6^A.^10^ FTO is broadly reported to exert important functions in mammalian development and diseases.^11^^,^^12^ *Fto* deficiency mice show marked growth retardation due to the critical roles of FTO in chromatin state regulation in mouse embryonic stem cells.^10^ In human cancer cells, FTO localizes to the cytoplasm and favors cancer progression.^13^^,^^14^^,^^15^^,^^16^ Moreover, FTO deficiency is involved in the development and aggravation of colitis.^17^ However, the functions of FTO in the context of OLP remain elusive.

Vitamin D is a pleiotropic hormone with a wide range of functions in humans.^18^^,^^19^ The activity of vitamin D is mediated by vitamin D receptor (VDR). Upon vitamin D treatment, the cellular VDR will move into the nucleus and tune the transcripts of downstream genes.^20^ Vitamin D/VDR signaling is closely related to the onset or development of OLP. Studies report that VDR expression in oral keratinocytes and vitamin D levels in serum are both decreased in OLP patients compared to healthy controls.^21^ At the molecular level, vitamin D/VDR signaling inhibits apoptosis and cytokine productions in keratinocytes from OLP through mediating microRNAs and inflammation-related factor levels.^22^^,^^23^^,^^24^^,^^25^ In this study, we suggested that FTO is deficient in the epithelial layer of OLP, and the lack of FTO facilitates epithelia dysfunction under inflammatory conditions.

## Results

### FTO protein levels are decreased in oral keratinocytes from OLP

**Figure 1:**
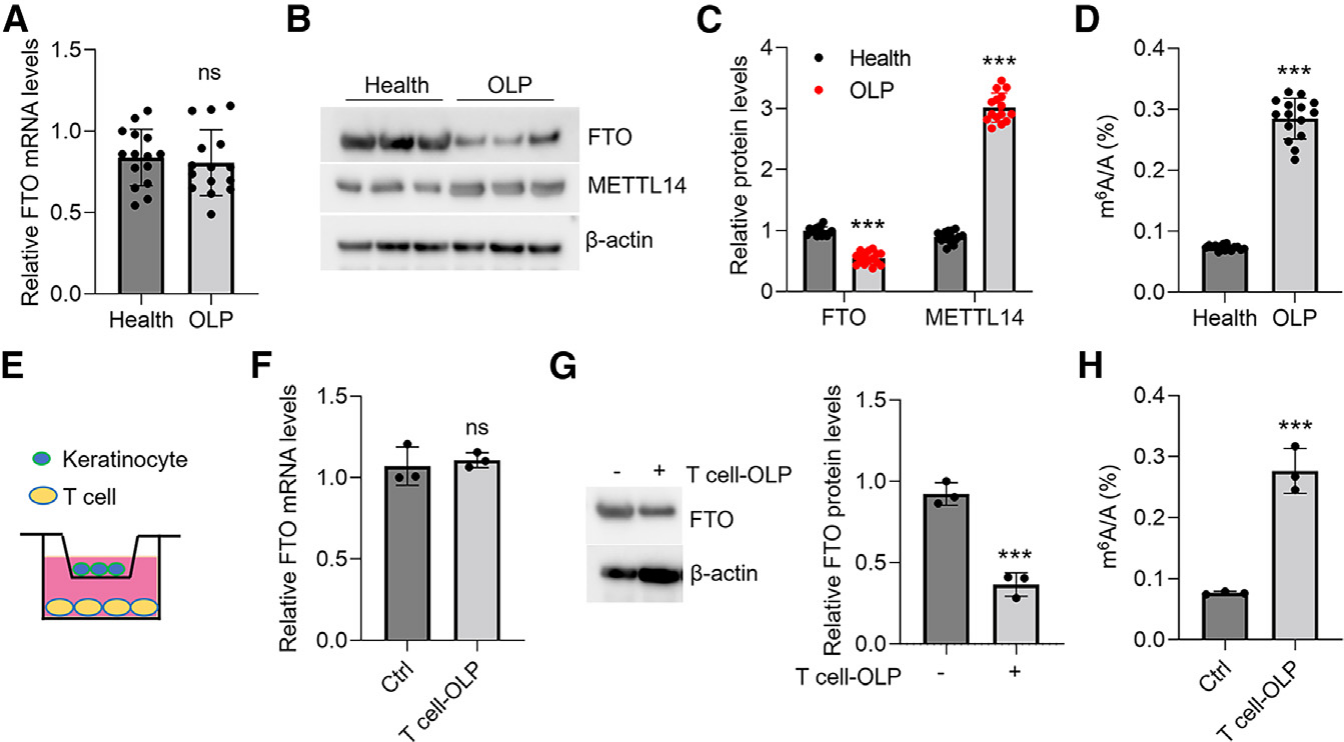
FTO protein levels are decreased in the oral keratinocytes from OLP (A) Real-time PCR showing *FTO* mRNA levels in the oral keratinocytes from healthy or OLP donors; *n* = 15. (B and C) FTO and METTL14 protein levels in the oral keratinocytes from healthy or OLP donors detected by western blot (B) and quantitative analysis (C); data are representative of 15 human samples in each group. (D) ELISA showing m^6^A levels in the oral keratinocytes from healthy or OLP donors; *n* = 15. (E) Schematic of oral mucosa-derived primary T cells and HOKs co-culture. HOKs were plated on the apical side and stimulated by the T cells in the basolateral chamber for 12 h. (F) Real-time PCR showing *FTO* mRNA levels of HOKs in the co-culture system; *n* = 3. (G) Western blot (left) and quantitative analysis (right) showing FTO protein levels of HOKs in the co-culture system; *n* = 3. (H) ELISA showing m^6^A levels of HOKs in the co-culture system; *n* = 3. ∗∗∗*p* < 0.001 vs. corresponding control group. Ctrl, control; OLP, oral lichen planus. Data are depicted as means ± standard deviations. Student’s t test was used for statistical analyses.

The status of FTO in oral keratinocytes in the setting of OLP remains unknown. To this end, we detected it by both qPCR and western blot. As shown, FTO protein levels, but not mRNA expression, were down-regulated in OLP-derived oral keratinocytes compared to healthy controls (Figures 1A–1C), along with the increase in m^6^A levels in OLP samples (Figure 1D). In line with our previous studies,^26^ we found that methyltransferase-like 14 (METTL14) protein levels were also elevated in OLP-derived oral keratinocytes (Figures 1B and 1C). Moreover, caspase-3 activity and cytokine expression in diseased oral keratinocytes were also elevated (Figures S1A and S1B), indicating the enhancement of apoptosis and inflammation. Interestingly, FTO protein levels showed negative correlations with caspase-3 activity and cytokine expression in OLP-derived oral keratinocytes (Figures S1C and S1D), implying that FTO might play a protective role in OLP development. Since the overactivation of T lymphocytes in oral mucosa primarily contributes to OLP initiation,^4^ we enriched oral mucosal primary T cells from healthy or OLP donors and co-cultured them with human oral keratinocytes (HOKs) to resemble OLP *in vitro* (Figure 1E). Consistently, HOKs co-cultured with OLP-derived primary T cells showed a decrease in FTO protein expression and an increase in m^6^A modification, along with elevated caspase-3 activity and cytokine production (Figures 1F–1H and S1E–S1G).

### GSK-3β promotes FTO protein degradation in oral keratinocytes from OLP

**Figure 2:**
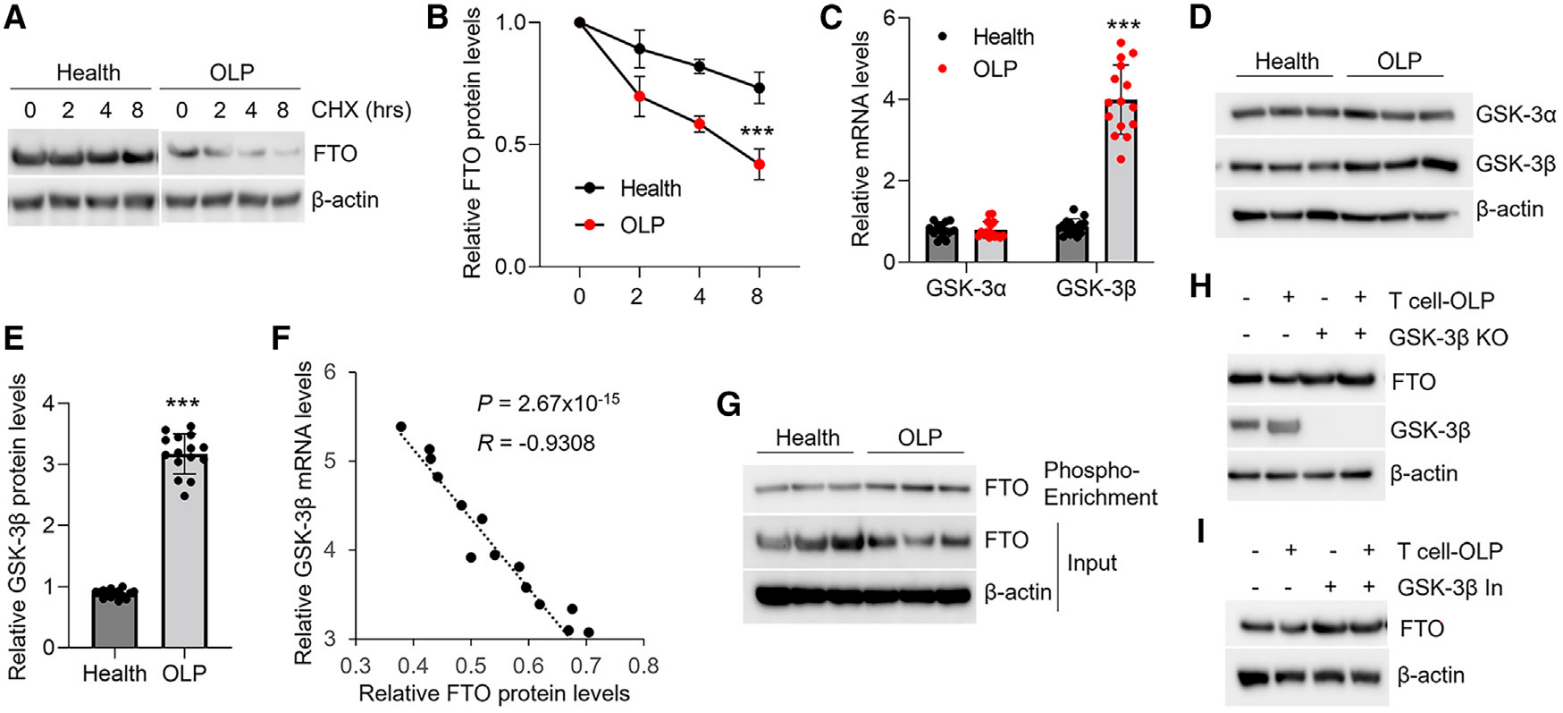
GSK-3β induces FTO protein degradation in the oral keratinocytes from OLP (A and B) Western blot data of FTO protein levels in the primary oral keratinocytes derived from healthy or OLP donors, with 50 μg/mL cycloheximide (CHX) treatment for distinct hours as indicated (A) and quantitative analyses (B); *n* = 3. (C) Real-time PCR showing GSK-3α and GSK-3β mRNA levels in the oral keratinocytes from healthy or OLP donors; *n* = 15. (D and E) Western blot data of GSK-3α and GSK-3β protein levels in the oral keratinocytes derived from healthy or OLP donors (D) and quantitative analyses (E); data are representative of 15 human samples. (F) Correlation analysis between FTO protein levels and *GSK-3β* mRNA expression in oral keratinocytes from OLP patients. (G) Western blot showing the FTO protein levels in the input or phosphor-enrichment samples of oral keratinocytes derived from healthy or OLP donors; data are representative of 15 human samples. (H and I) Western blot analyses of FTO protein expression in the GSK-3β lentivirus-transduced (H) or GSK-3β inhibitor-treated (I) HOKs co-cultured with or without OLP-derived primary T cells; data are representative of three independent biological experiments. ∗∗∗*p* < 0.001 vs. corresponding control group. KO, knockout. Data are depicted as means ± standard deviations. Two-way ANOVA (B) and Student’s t test (C, E, and F) were used for statistical analysis.

To explain the molecular mechanism by which FTO protein is decreased, we isolated primary oral keratinocytes from healthy and OLP donors and cultured them with cycloheximide (CHX) for protein decay assays. As expected, protein degradation was enhanced in the diseased oral keratinocytes (Figures 2A, 2B, and S2A). Glycogen synthase kinase-3β (GSK-3β) is reported to induce FTO protein decay in mouse embryonic stem cells.^27^ Here, we found that GSK-3β expression was increased in the diseased oral keratinocytes rather than GSK-3α (Figures 2C–2E). Moreover, GSK-3β expression was negatively correlated with FTO protein levels in the diseased oral keratinocytes (Figure 2F). This finding was also confirmed in the co-culture model (Figures S2B and S2C). In line with previous studies,^27^ FTO phosphorylation in OLP-derived oral keratinocytes was increased (Figure 2G). Genetic ablation or pharmacological inhibition of GSK-3β prevented FTO protein decreases in the cell model (Figures 2H and 2I).

Phosphorylation on serines 249 and 253 is reported to regulate murine FTO protein degradation.^27^ By using the online sequence alignment tool (https://en.vectorbuilder.com/tool/sequence-alignment.html), we found that phosphorylation on serine 256 might affect human FTO protein decay. To this end, we generated human FTO in which serine 256 was mutated to alanine (FTO-S256A). As shown, mutation on serine 256 could block human FTO protein degradation (Figure S3A). Overexpression of GSK-3β induced FTO phosphorylation and decreased FTO protein levels in HOKs (Figure S3B), while mutation on serine 256 reversed the effects of GSK-3β on FTO (Figure S3C). These data indicated that GSK-3β regulates FTO protein degradation via serine 256 phosphorylation in HOKs.

### FTO deficiency facilitates oral keratinocyte dysfunction in the OLP cell model

**Figure 3:**
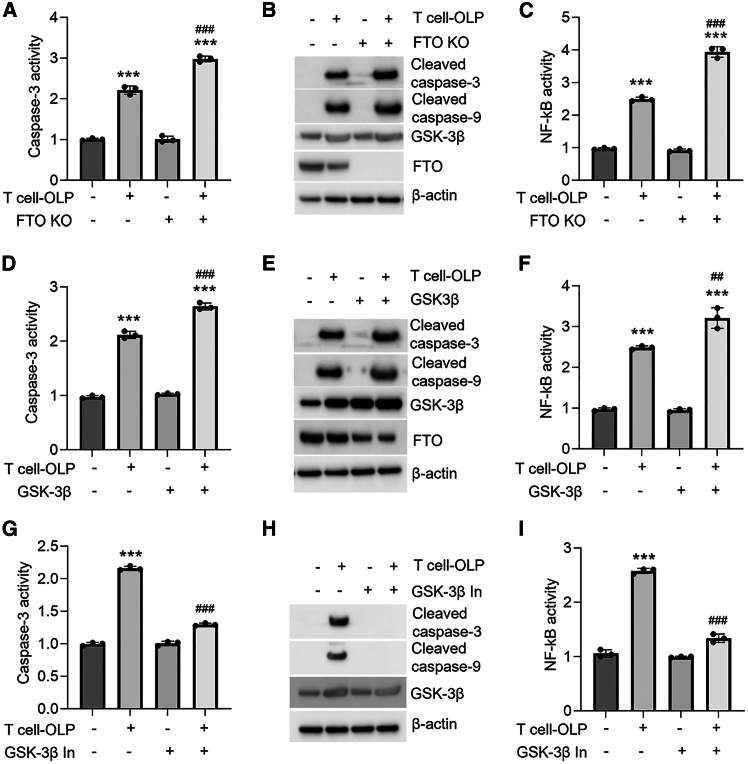
FTO deficiency facilitates OLP development (A–C) Caspase-3 activity (A), protein levels (B), or NF-κB activity (C) in primary T cell-co-cultured HOKs with or without FTO knockout. (D–F) Caspase-3 activity (D), protein levels (E), or NF-κB activity (F) in primary T cell-co-cultured HOKs with or without GSK-3β overexpression. (G–I) Caspase-3 activity (G), protein levels (H), or NF-κB activity (I) in primary T cell-co-cultured HOKs with or without 12-h GSK-3β inhibitor treatment. *n* = 3; western blot data are representative of three independent biological experiments. ∗∗∗*p* < 0.001 vs. corresponding control group; ##*p* < 0.01; ###*p* < 0.001 vs. T cell-OLP group. In, inhibitor. Data are depicted as means ± standard deviations. One-way ANOVA was used for statistical analysis.

To explore the role of FTO in the development of OLP, we deleted the *FTO* gene of HOKs prior to co-culture. As shown, the loss of FTO facilitated apoptosis and nuclear factor κB (NF-κB) activity in HOKs co-cultured with OLP-derived primary T cells (Figures 3A–3C). Similar results were observed in HOKs overexpressing GSK-3β in the co-culture system (Figures 3D–3F). Conversely, GSK-3β inhibitor, which could promote FTO expression, ameliorated primary T cell-stimulated HOK damage (Figures 3G–3I).

### FTO regulates VDR transcripts in an m^6^A-dependent manner

**Figure 4:**
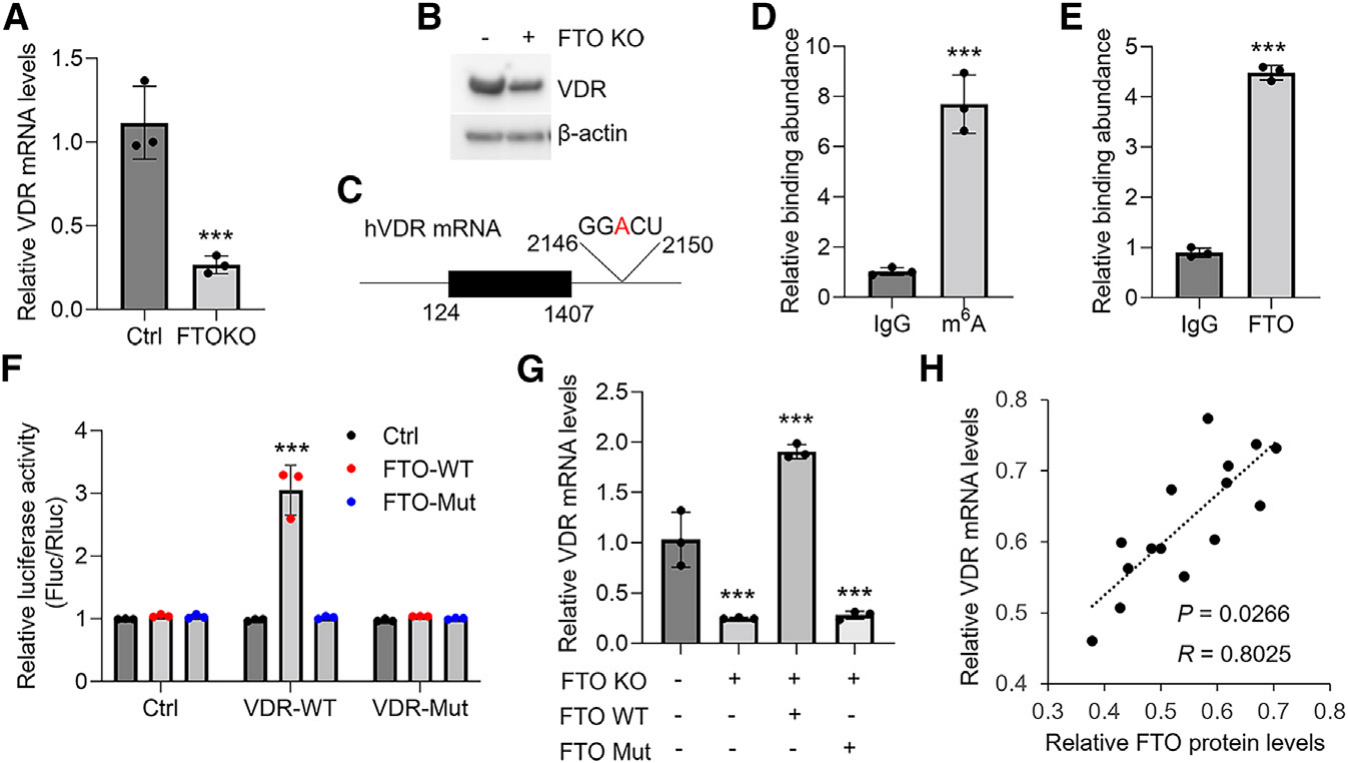
FTO regulates VDR expression in HOKs in an m^6^A-dependent way (A and B) Real-time PCR (A) and western blot (B) showing VDR levels in HOKs upon FTO knockout. (C) Schematic showing the m^6^A motif in the human *VDR* mRNA. (D) RIP-qPCR showing the m^6^A peak of *VDR* transcript in HOKs using anti-immunoglobulin G (IgG) or anti-m^6^A antibodies. (E) CLIP-qPCR showing the binding of FTO to the VDR m^6^A site in HOKs using anti-IgG or anti-FTO antibodies. (F) Luciferase report assays demonstrating the roles of WT or mutated FTO in WT or mutated VDR reporters. (G) Real-time PCR showing *VDR* mRNA levels in FTO knockout HOKs with WT or mutated FTO overexpression. (H) Correlation analysis between FTO protein levels and VDR mRNA expression in OLP oral keratinocytes; *n* = 3. Western blot data are representative of three independent biological experiments. ∗∗∗*p* < 0.001 vs. corresponding control group. Mut, mutated; WT, wild type. Data are depicted as means ± standard deviations. Student’s t test (A, D, E, and H), two-way ANOVA (F), and one-way ANOVA (G) were used for statistical analysis.

Our previous data have shown that VDR exerts critical protective functions in OLP development.^23^^,^^24^^,^^25^ To answer whether FTO is able to mediate VDR expression in HOKs, we assessed VDR levels in HOKs upon *FTO* knockout. As displayed, both mRNA and protein levels of VDR were decreased in HOKs upon *FTO* deletion, accompanied by the elevation of m^6^A status (Figures 4A, 4B, and S4A). We next investigated the human *VDR* mRNA and found an m^6^A site (Figure 4C) using the online software Cuilab (https://www.cuilab.cn/sramp). RNA immunoprecipitation (RIP)-qPCR and cross-linking and immunoprecipitation (CLIP)-qPCR assays verified the bindings of m^6^A modification as well as FTO to this m^6^A motif in human *VDR* mRNA (Figures 4D and 4E). Luciferase report assays indicated that FTO, but not the demethylase-inactive mutant, enhanced luciferase activity in reporters containing wild-type (WT) VDR fragments but not m^6^A site-mutated fragments (Figure 4F). The forced expression of WT FTO rather than demethylase-inactive mutant reversed VDR decreases in HOKs upon endogenous *FTO* knockout (Figure 4G). In the context of OLP, FTO protein levels showed a positive correlation with VDR mRNA expression in diseased oral keratinocytes (Figure 4H). Along with FTO down-regulation (Figure 1G), OLP-derived primary T cells also could decrease VDR expression in HOKs (Figures S4B and S4C). GSK-3β overexpression, which is able to decrease FTO expression, down-regulated VDR levels in HOKs, while GSK-3β inhibitor had a inverse effect (Figures S4D and S4E).

### FTO deficiency facilitates oral keratinocyte damage in a VDR-dependent way

**Figure 5:**
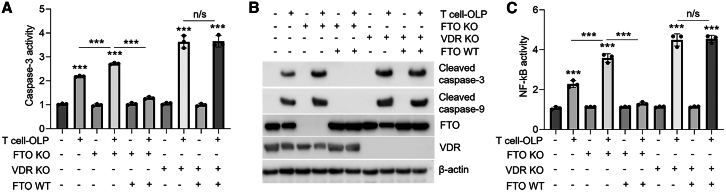
FTO plays a role in OLP progression dependent on VDR Caspase-3 activity (A), cleaved caspase-3 and cleaved caspase-9 expression (B), or NF-κB activity (C) in primary T cell-co-cultured HOKs with or without lentivirus infection as indicated; *n* = 3. Western blot data are representative of three independent biological experiments. ∗∗∗*p* < 0.001 vs. corresponding control group. Data are depicted as means ± standard deviations. One-way ANOVA was used for statistical analysis.

To further answer whether FTO deficiency depends on VDR to damage oral keratinocytes, we knocked out *VDR* gene in HOKs. In the presence of endogenous VDR, FTO overexpression could rescue primary T cell-induced oral keratinocyte damage. However, forced expression of FTO failed to suppress primary T cell-induced apoptosis and NF-κB activity in HOKs upon VDR depletion (Figures 5A–5C). Meanwhile, GSK-3β inhibitor was unable to overcome the overactivation of apoptosis and NF-κB activity in HOKs upon VDR knockout (Figures S5A–S5C).

### Targeting RNA m^6^A methylation regulates the dysfunction of oral keratinocytes

**Figure 6:**
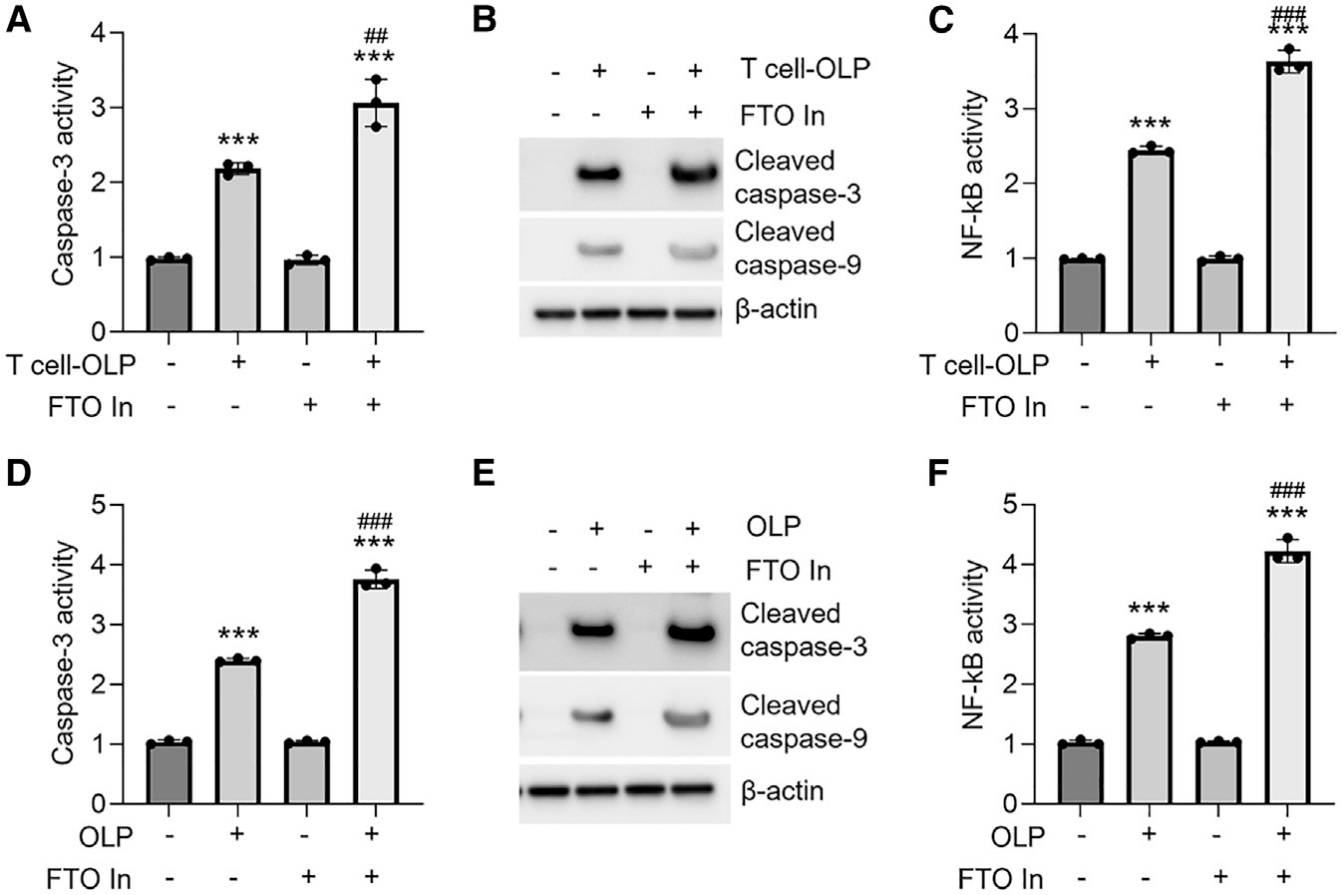
The effects of FTO inhibitor on OLP (A–C) Caspase-3 activity (A), cleaved caspase-3 and cleaved caspase-9 expression (B), or NF-κB activity (C) in primary T cell-co-cultured HOKs with or without 12-h FTO inhibitor treatment. (D–F) Caspase-3 activity (D), cleaved caspase-3 and cleaved caspase-9 expression (E), or NF-κB activity (F) in human oral mucosa-derived primary keratinocytes with or without 12-h FTO inhibitor treatment; *n* = 3. Western blot data are representative of three independent biological experiments. ∗∗∗*p* < 0.001 vs. corresponding control group; ##*p* < 0.01; ###*p* < 0.001 vs. T cell-OLP or OLP group. Data are depicted as means ± standard deviations. One-way ANOVA was used for statistical analysis.

Since FTO has critical regulatory roles in OLP development, we reasoned that mediation of RNA m^6^A methylation might affect the progression of OLP. In the presence of FTO inhibitor treatment, apoptosis and NF-κB activity overactivation were both enhanced in HOKs upon OLP-derived primary T cell stimulation (Figures 6A–6C). We next isolated primary oral keratinocytes from healthy and OLP donors and cultured them with or without FTO inhibitor treatment. As shown, FTO inhibitor also elevated apoptosis and NF-κB activity in primary cells from OLP (Figures 6D–6F). On the contrary, METTL3 inhibitor, which is able to decrease m^6^A modifications, protects oral keratinocytes from damage (Figures S6A–S6F). These results suggest that regulation of RNA m^6^A methylation might be an effective approach for OLP management.

## Discussion

FTO, which is responsible for the regulation of RNA m^6^A methylation, is associated with autoimmune disease development.^17^ In this investigation, we identified that the expression of FTO protein, but not mRNA, was decreased in the keratinocytes derived from OLP tissues in comparison to healthy controls. Consistent with our previous investigations,^26^ we confirmed that METTL14 levels were increased in the keratinocytes derived from OLP tissues. Therefore, both FTO decrease and METTL14 increase contribute to the elevated m^6^A levels in diseased oral samples. In line with previous studies,^27^ we confirmed that the decrease in FTO in diseased oral keratinocytes was due to the enhanced FTO protein degradation induced by GSK-3β. In addition, GSK-3β was also up-regulated in the keratinocytes from OLP, indicating that GSK-3β inhibitor might be a novel therapeutic strategy for the treatment of OLP.

So far, there is no well-established cell or animal model resembling OLP. Since OLP is known as a T lymphocyte-triggered inflammatory disease,^4^ we isolated primary T cells from diseased oral mucosae and co-cultured them with HOKs to mimic OLP *in vitro*. In agreement with the data concerning human samples, we found that FTO levels were decreased while GSK-3β expression was increased in the HOKs co-cultured with primary T cells from OLP. Although this is not a perfect cell model as other kinds of immune cells such as macrophages or dendritic cells are also involved in the onset and development of OLP, the consistent data from both human samples and the cell model suggest that this co-culture system works well for mimicking. As CD8^+^ T cells play critical roles in the degeneration of oral keratinocytes,^28^ we purified all T cells from oral mucosae for co-culture rather than CD4^+^ T cells.

The degeneration of keratinocytes is a major outcome of uncontrolled immune reactions in OLP.^28^ Here, we verified that FTO deficiency facilitated oral epithelia dysfunction in the context of OLP, consistent with other studies showing that FTO insufficiency aggravates ulcerative colitis.^17^ Under inflammatory conditions, the lack of FTO promoted NF-κB activity and apoptosis in oral keratinocytes. On a molecular basis, FTO impacted oral keratinocytes degeneration by targeting VDR. Vitamin D/VDR signaling is reported to play a protective role in the progression of OLP,^21^^,^^22^^,^^23^ but the molecular underpinning of VDR decrease in oral keratinocytes from OLP is not fully explained. In this study, we found that FTO deficiency diminished VDR mRNA levels in an m^6^A-dependent manner in HOKs, implying that RNA m^6^A methylation exerts important functions in OLP development. Moreover, since vitamin D/VDR signaling can inactivate the NF-κB pathway to inhibit cell apoptosis in the context of OLP,^22^ NF-κB activation and apoptosis might be sequentially regulated by GSK-3β-FTO-VDR axis in oral keratinocytes.

Other studies have suggested that m^6^A methylation of Socs1 is required to suppress inflammatory responses in murine macrophages upon bacterial infection.^29^ Given that vitamin D/VDR signaling is reported to up-regulate SOCS1 levels in mouse macrophages,^30^ investigations regarding the effects of VDR or m^6^A on SOCS1 in HOKs under OLP conditions would be very interesting.

Combined with our previous studies noting that METTL14 regulates the cell death of keratinocytes in OLP,^26^ we propose that targeting RNA m^6^A methylation may be an effective therapeutic means for OLP management. More basic and clinical experiments are required to verify this hypothesis in the future.

## Materials and methods

### Human samples

Human oral tissues were obtained from healthy donors and OLP patients at the Stomatological Hospital of Shanxi Medical University. The OLP inclusion and exclusion rules were followed according to the modified World Health Organization diagnostic criteria. Control healthy samples were from people who underwent third molar extractions. This human study was approved by the Ethical Committee of Shanxi Medical University (no. 2016LL046). Informed consent was received from all participants. More details for individuals in this study are listed in Table S1.

### Cell culture

HOKs (ScienCell, catalog no. 2610) were cultured with oral keratinocyte medium supplemented with 1% penicillin/streptomycin and 10% fetal bovine serum (FBS) under 37°C and 5% CO_2_ conditions. In separate experiments, GSK-3 inhibitor SB-415,286 (10 μM), METTL3 inhibitor STM2457 (5 μM), or FTO inhibitor FTO-04 (20 μM) was supplemented into keratinocyte culture media for 12 h prior to T cell-HOK co-culture. Transwell inserts (0.4 μm pore size; Corning, catalog no. 3470) were used for the primary T cell and HOK co-culture system, in which HOKs were stimulated by T cells for 12 h.

### Primary oral epithelial cells and T cell isolation

The fresh human oral mucosae were treated with 0.25% dispase II for dissociation, followed by the separation of the oral mucosal epithelial layer by muscle forceps, as mentioned.^22^ Both the epithelial layer and the lamina propria tissue were dissociated into single cells using the Papain Dissociation System (Worthington, catalog no. LK003150). Primary human T cells from lamina propria were enriched by the Pan T cell Isolation Kit (Miltenyi Biotechnology, catalog no. 130-096-535).

### Lentiviral or plasmid construction

Lentivirus expressing human FTO or GSK-3β was generated by cloning the cDNA of FTO (NM_001363894.1) or GSK-3β (NM_002093.4) into pLV[Exp]-Neo-EF1A lentiviral backbone from VectorBuilder. Single-guide RNA (sgRNA) sequences targeting the FTO gene (5′-AGCTTCGCGCTCTCGTTCCT-3′), GSK-3β gene (5′-AGATGAGGTCTATCTTAATC-3′), or VDR gene (5′-ACGTTCCGGTCAAAGTCTCC-3′) were inserted into lentiCRISPRv2 vector (Addgene, catalog no. 52961) for FTO, GSK-3β, or VDR ablation. Lentiviral vectors with cDNAs or sgRNAs were transfected into HEK293T for packaging. Cell culture medium with lentivirus was collected 48 h later and used to infect cells. The fragment containing m^6^A motif in VDR cDNA was cloned into the Luc gene in pGL3-Promoter (Promega, catalog no. 200517) for the pGL3-VDR plasmid construction. The pGL3-VDR-Mut was made by changing “A” to “T” in the m^6^A motif via a QuickChange Site-Directed Mutagenesis Kit (Agilent, catalog no. 200523). All of the plasmids were verified by DNA sequencing. Related primers are listed in Table S2.

### m^6^A quantitation

The m^6^A quantitation analysis was performed using a commercial m^6^A ELISA kit (Epigentek, catalog no. P-9005-48) following the manufacturer’s protocol as described.^31^

### NF-κB activity

NF-κB activity was detected using the commercial NF-κB luciferase reporter kit (BPS Biosci-ence, catalog no. 60614) per the manufacturer’s instructions. In brief, keratinocytes were co-transfected with Renilla luciferase reporter and NF-κB luciferase reporter. The activities of luciferase were measured using a Dual Luciferase System (Berthold Technologies).

### Caspase-3 activity

Caspase-3 activity was detected using the commercial Caspase-3 Assay Kit (Abcam, catalog no. ab39401) per the manufacturer’s instructions.

### Luciferase reporter experiment

The luciferase reporter experiments were performed as described.^31^ Briefly, cells were seeded onto 48-well plates prior to the 48-h lentivirus infection. Cells were then co-transfected with 500 ng pGL3-promoter, pGL3-VDR, or pGL3-VDR-Mut plasmids and pRL-TK Renilla luciferase reporter for 24 h. The activities of luciferase were tested using a Luciferase Assay System kit (Promega, catalog no. G7941).

### Western blot

Tissues or cells were homogenized in radioimmunoprecipitation assay buffer with protease inhibitor cocktail (Roche). The Pierce BCA Protein Assay Kit (Thermo Fisher Scientific) was used to determine protein concentration. Lysates were heated at 95°C for 10 min to denature proteins. We loaded 30 μg proteins into the wells of SDS-PAGE gel and then transferred them to the polyvinylidene fluoride membrane (Millipore), which was further treated with 5% BSA and incubated with primary antibodies at 4°C overnight. On day 2, the membrane was incubated with horseradish peroxidase-conjugated secondary antibodies at room temperature for 1 h. Protein signals were detected by the Pierce ECL Western Blotting Substrate (Thermo Fisher Scientific) and quantified by ImageJ software (NIH). The details of the primary antibodies are listed in Table S3.

### RT-qPCR, MeRIP-qPCR and CLIP-qPCR for RNA samples

Total RNAs from tissues or cells were isolated by TRIzol Reagent (Thermo Fisher Scientific) and reverse transcribed using the ReverTra Ace qPCR RT Kit (TOYOBO). Real-time qPCR was performed using the SYBR Green Realtime PCR Master Mix kit (TOYOBO). Glyceraldehyde 3-phosphate dehydrogenase was used for the internal control. Methylated (Me)RIP-qPCR was carried out using the EpiMark *N*^6^-Methyladenosine Enrichment Kit (NEB) following the manufacturer’s protocols, as described before.^29^ CLIP assays were conducted as described previously, with some modifications.^29^ In brief, HOKs were cross-linked with UV irradiation on ice, followed by collection with lysis buffer. FTO antibodies were conjugated with Protein A/G Magnetic Beads (Thermo Fisher Scientific) to precipitate target mRNAs. Immunoprecipitated RNAs were then harvested with TRIzol reagents and subjected to qPCR. The primers are listed in Table S2.

### Statistical analysis

Data were described as means ± standard deviations. Two groups’ comparisons were performed using unpaired two-tailed Student’s t tests. Three or more groups’ comparisons were performed with one-way or two-way ANOVA. *p* < 0.05 was identified as statistically significant.

## Data availability

The data that support the findings of this study are available from the corresponding author upon reasonable request.

## Acknowledgments

This work was supported by the Natural Science Foundation of Shanxi (grant nos. 202303021211127, 202103021224231, and 202302020101009), the Shanxi Provincial Key Research and Development Plan Project (grant no. 202302020101009), the Hainan Province Health Science and Technology Innovation Joint Project (grant no. WSJK2024MS237), The Excellent Talent Team of Hainan Province (grant no. QRCBT202121), and the Hainan Province Clinical Medical Center.

## Author contributions

X.G. conceived and designed the research. Y.F., Y.H., Y.D., and X.W. performed the experiments. X.W. provided assistant work for the experiments. Y.F. collected human samples and analyzed the data. X.G. supervised the patient research and wrote the manuscript. X.G. and X.W. acquired the funding. All authors read and approved the final manuscript.

## Declaration of interests

The authors declare no competing interests.

## Supplemental information

Document S1. Figures S1–S6 and Tables S1–S3

Document S2. Article plus supplemental information
