# Supplementary material for: FTO deficiency facilitates epithelia dysfunction in oral lichen planus
Source: Mol Ther Nucleic Acids. 2025 Jan 25;36(1):102463. doi: 10.1016/j.omtn.2025.102463 (PMC11847738; doi:10.1016/j.omtn.2025.102463)
Supplement: Document S1. Figures S1–S6 and Tables S1–S3 [file mmc1.pdf]

**OMTN, Volume *36***

## Supplemental information

# FTO deficiency facilitates epithelia dysfunction in oral lichen planus

**Yufeng Fan, Yukai Hao, Yan Ding, Xiangyu Wang, and Xuejun Ge**

## Supplementary Figures

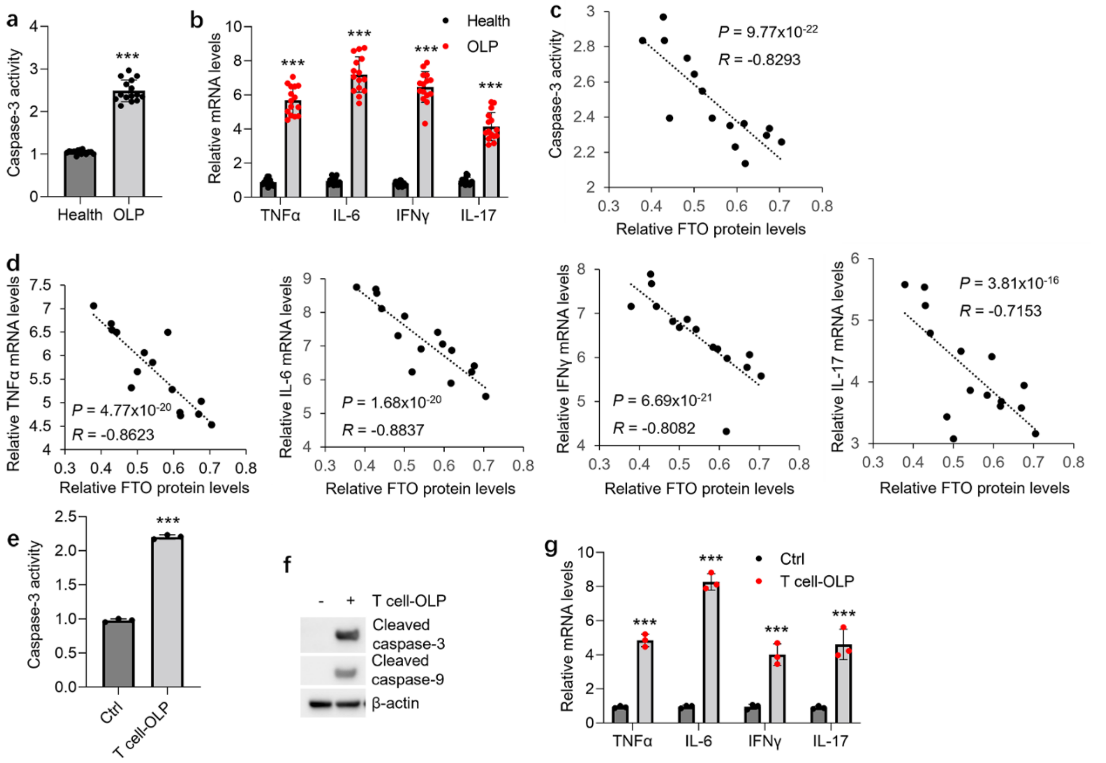


Figure S1. Apoptosis and cytokines levels in OLP. (a) Caspase 3 activity in the oral keratinocytes from healthy or OLP donors, n = 15. (b) Real-time PCR demonstrating cytokines expression in the oral keratinocytes from healthy or OLP donors, n = 15. (c-d) Correlation analyses between FTO protein levels and caspase 3 activity (c) or cytokines expression (d) in the oral keratinocytes from OLP patients. (e-g) Caspase 3 activity (e), cleaved caspase 3 and cleaved caspase 9 levels (f) or cytokines expression (g) of HOKs in the co-culture model, data are representative of 3 independent biological experiments. *** $P < 0.001$ vs corresponding control group; OLP, oral lichen planus; Ctrl, control. Data were depicted as means ± standard deviation. Student's *t* test was used for statistical analysis.

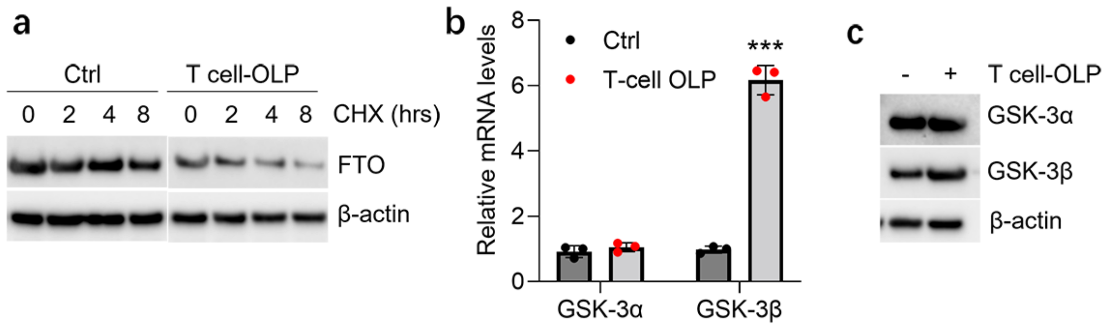


Figure S2. FTO protein decay in the co-culture model. (a) Western blot data of FTO protein levels in HOKs co-cultured with or without primary T cells derived from OLP donors. HOKs were treated with 50 µg/ml cycloheximide (CHX) for distinct hours as indicated prior to co-culture. (b) Real-time PCR showing *GSK-3α* and *GSK-3β* mRNA levels in HOKs co-cultured with or without OLP-derived primary T cells, n = 3. (c) Western blot showing GSK-3α and GSK-3β protein levels in HOKs co-cultured with or without OLP-derived primary T cells. Western blot data are representative of 3 independent biological experiments. *** $P < 0.001$ vs corresponding control group; OLP, oral lichen planus; Ctrl, control. Data were depicted as means ± standard deviation. Student's *t* test was used for statistical analysis.

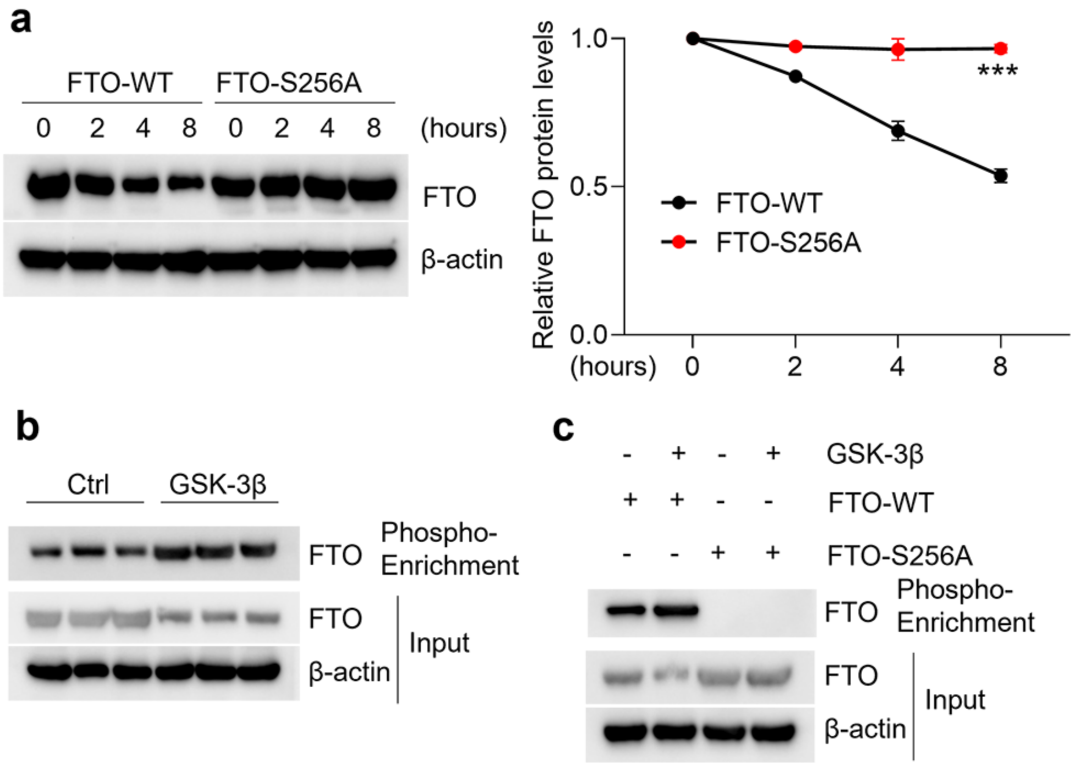


Figure S3. Analysis of FTO phosphorylation and degradation. (a) The endogenous *FTO* gene in HOKs was deleted, and then FTO-deleted HOKs were infected with lentivirus overexpressing FTO-WT or FTO-S256A. Western blot data (left) and quantitative analysis (right) of FTO protein levels in HOKs which were treated with 50 µg/ml cycloheximide (CHX) for distinct hours as indicated after infection. (b) Western blot showing the FTO protein levels in the input or phosphor-enrichment samples of HOKs with or without GSK-3β overexpression. (c) The endogenous *FTO* gene in HOKs was deleted, and then FTO-deleted HOKs were infected with GSK-3β, FTO-WT or FTO-S256A lentivirus as indicated. Western blot showing the FTO protein levels in the input or phosphor-enrichment samples of HOKs after infection. n = 3, western blot data are representative of 3 independent biological experiments. *** $P < 0.001$ vs corresponding control group. Two-way ANOVA was used for statistical analysis.

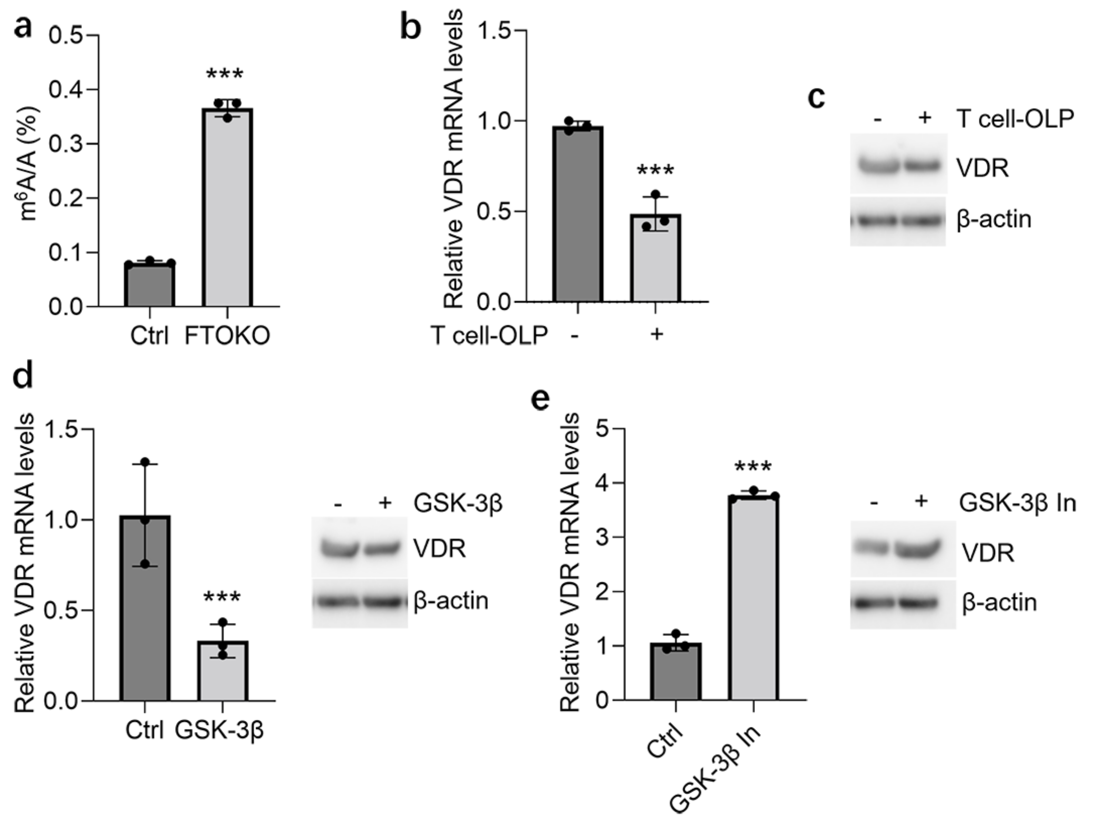


Figure S4. The roles of GSK-3β in VDR expression. (a) m[6]A levels of HOKs with or without FTO knockout. (b-c) Real-time PCR (b) or Western blot (c) showing VDR levels in HOKs co-cultured with primary T cells. (d) VDR levels in HOKs with or without GSK-3β overexpression detected by qPCR (left) or western blot (right). (e) VDR levels in HOKs with or without GSK-3β inhibitor treatment detected by qPCR (left) or western blot (right). n = 3, western blot data are representative of 3 independent biological experiments. *** $P < 0.001$ vs corresponding control group; Ctrl, control; KO, knockout; In, inhibitor. Data were depicted as means ± standard deviation. Student's *t* test was used for statistical analysis.

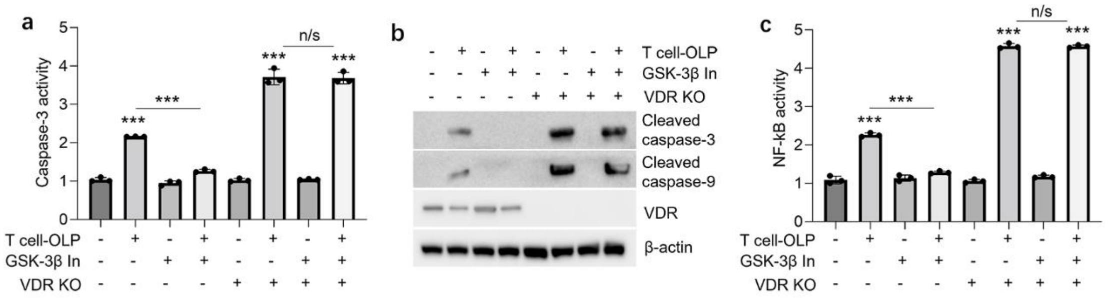


Figure S5. Roles of VDR in OLP development. (a-c) Caspase 3 activity (a), cleaved caspase 3 and cleaved caspase 9 expression (b), or NF-κB activity (c) in VDR knockout HOKs with or without primary T cell co-culture or 12-hour GSK-3β inhibitor treatment. n = 3, western blot data are representative of 3 independent biological experiments. *** $P < 0.001$ vs corresponding control group; KO, knockout; In, inhibitor. Data were depicted as means ± standard deviation. One-way ANOVA was used for statistical analysis.

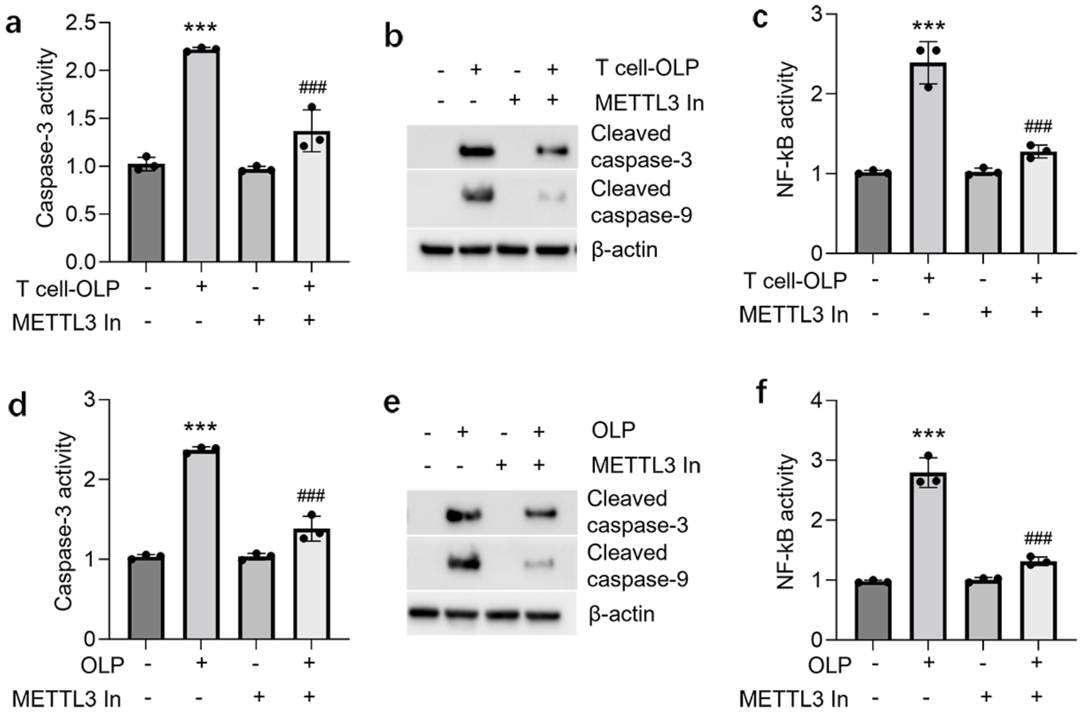


Figure S6. The effects of METTL3 inhibitor on OLP. (a-c) Caspase 3 activity (a), cleaved caspase 3 and cleaved caspase 9 expression (b), or NF-κB activity (c) in primary T cell-co-cultured HOKs with or without 12-hour METTL3 inhibitor treatment. (d-f) Caspase 3 activity (d), cleaved caspase 3 and cleaved caspase 9 expression (e), or NF-κB activity (f) in human oral mucosa-derived primary keratinocytes with or without 12-hour METTL3 inhibitor treatment. n = 3, western blot data are representative of 3 independent biological experiments. *** $P < 0.001$ vs corresponding control group; ## $P < 0.01$, ### $P < 0.001$ vs T cell-OLP or OLP group; In, inhibitor. Data were depicted as means ± standard deviation. One-way ANOVA was used for statistical analysis.

Table S1

Clinical parameters of OLP patients in this study

| No. | Age (year) | Sex | Site | Subtype |
|---|---|---|---|---|
| 1 | 64 | Male | Buccal | Reticular |
| 2 | 45 | Male | Buccal | Reticular |
| 3 | 58 | Female | Buccal | Reticular |
| 4 | 43 | Female | Buccal | Reticular |
| 5 | 40 | Female | Buccal | Reticular |
| 6 | 63 | Male | Buccal | Reticular |
| 7 | 37 | Male | Buccal | Reticular |
| 8 | 52 | Male | Buccal | Reticular |
| 9 | 49 | Female | Buccal | Reticular |
| 10 | 32 | Female | Buccal | Reticular |
| 11 | 38 | Female | Buccal | Reticular |
| 12 | 45 | Female | Buccal | Reticular |
| 13 | 48 | Male | Buccal | Reticular |
| 14 | 50 | Male | Buccal | Reticular |
| 15 | 60 | Female | Buccal | Reticular |

Table S2

Sequences of primers involved in this study

| Name | Forward 5'-3' | Reverse 5'-3' |
|---|---|---|
| qPCR primers | | |
| hFTO | ACTTGGCTCCCTTATCTGACC | TGTGCAGTGTGAGAAAGGCTT |
| hTNFα | CGAGTGACAAGCCTGTAGC | GGTGTGGGTGAGGAGCACAT |
| hIL-6 | TGAGGAGACTTGCCTGGTGA | GTTGGGTCAGGGGTGGTTAT |
| hIFNγ | TGAACATGATGGATCGTTGG | CATTCACTTTGCTGGCAGTG |
| hIL-17 | TCCCACGAAATCCAGGATGC | GGATGTTCAGGTTGACCATCAC |
| hGSK-3α | GGAAAGGCATCTGTCGGGG | GAGTGGCTACGACTGTGGTC |
| hGSK-3β | GGCAGCATGAAAGTTAGCAGA | GGCGACCAGTTCTCCTGAATC |
| hVDR | GACTTTGACCGGAACGTGCCC | CATCATGCCGATGTCCACACA |
| hGAPDH | ACCACAGTCCATGCCATCAC | TCCACCACCCTGTTGCTGTA |
| hVDR $m^6A$ | CCCTGTCACCAAGCTCACAG | AGGCACTGGCAGGGGGAGGA |
| Lentivirus and plasmids construction primers | | |
| hFTO cDNA | ATGAAGCGCACCCCGACTGCCGA | CTAGGGTTTTGCTTCCAGAAGCT |
| hFTO R316Q | ACAACCTCAGTTTAGTTCCA | GAACCGGCCAAAACACAGTG |
| hFTO R322Q | TTTAGTTCCACCCACCAAGT | CTGAGGTTGTGAACCGGCCA |
| hFTO S256A | AGAGGAAGCTGAGGATGACT | TCAGGGCCTTCACAGCTATA |
| hGSK-3β cDNA | ATGTCAGGGCGGCCCAGAACCACCT | TCAGGTGGAGTTGGAAGCTGAT |
| pGL3-VDR | TGGGTCTAAGGGGTTGGTTG | AGGCACTGGCAGGGGGAGG |
| pGL3-VDR-mut | TAGCAGGCCTGAATTGTCCC | CATGGATCCGTGGAAGGAGG |

Table S3

Antibodies information of this study

| Antibodies | SOURCE | IDENTIFIER |
|---|---|---|
| Anti-VDR | Santa Cruz Biotechnology | Cat#: sc-13133, RRID: AB_628040 |
| Anti-β-actin | Santa Cruz Biotechnology | Cat#: sc-47778, RRID: AB_2714189 |
| Anti-FTO | Abcam | Cat#: ab92821, RRID: AB_10565042 |
| Anti-cleaved caspase 3 | Cell Signaling Technology | Cat#: 9664S, RRID:AB_2070042 |
| Anti-cleaved caspase 9 | Cell Signaling Technology | Cat#: 9505, RRID:AB_2290727 |
| Anti-GSK-3α | ProteinTech | Cat#: 13419-1-AP, RRID:AB_2247995 |
| Anti-GSK-3β | ProteinTech | Cat#: 22104-1-AP, RRID:AB_2878997 |
| Anti-m6A | Synaptic Systems | Cat#: 202003, RRID: AB_2279214 |
